# Shoulder Mobility, Muscular Strength, and Quality of Life in Breast Cancer Survivors with and without Tai Chi Qigong Training

**DOI:** 10.1155/2013/787169

**Published:** 2013-04-23

**Authors:** Shirley S. M. Fong, Shamay S. M. Ng, W. S. Luk, Joanne W. Y. Chung, Louisa M. Y. Chung, William W. N. Tsang, Lina P. Y. Chow

**Affiliations:** ^1^Institute of Human Performance, The University of Hong Kong, Pokfulam, Hong Kong; ^2^Department of Rehabilitation Sciences, The Hong Kong Polytechnic University, Hong Kong; ^3^The Association of Licentiates of Medical Council of Hong Kong, Hong Kong; ^4^Department of Health and Physical Education, The Hong Kong Institute of Education, Hong Kong

## Abstract

*Objectives*. To compare the shoulder mobility, muscular strength, and quality of life (QOL) among breast cancer survivors with and without Tai Chi (TC) Qigong training to those of healthy individuals and to explore the associations between shoulder impairments and QOL in breast cancer survivors with regular TC Qigong training. *Methods*. Eleven breast cancer survivors with regular TC Qigong training, 12 sedentary breast cancer survivors, and 16 healthy participants completed the study. Shoulder mobility and rotator muscle strength were assessed by goniometry and isokinetic dynamometer, respectively. QOL was assessed using the Functional Assessment of Cancer Therapy-Breast (FACT-B) questionnaire. *Results*. Goniometric measurements of the active range of motion in the flexion, abduction, and hand-behind-the-back directions were similar among the three groups. The TC Qigong-trained breast cancer survivors had significantly higher isokinetic peak torques of the shoulder rotator muscles (at 180°/s) than untrained survivors, and their isokinetic shoulder muscular strength reached the level of healthy individuals. Greater shoulder muscular strength was significantly associated with better functional wellbeing in breast cancer survivors with TC Qigong training. However, no significant between-group difference was found in FACT-B total scores. *Conclusions*. TC Qigong training might improve shoulder muscular strength and functional wellbeing in breast cancer survivors.

## 1. Introduction

Breast cancer (CA) is the most common malignant disease affecting women of all ages [1]. Despite the increased survival rates of breast CA patients [1], conventional treatments (e.g., mastectomy and radiotherapy) often result in side effects such as impaired shoulder function with decrements in shoulder muscular strength, shoulder mobility, and functional capacity [2, 3]. These side effects may persist or even increase several years posttreatment and are often severe enough to reduce self-esteem and diminish the quality of life (QOL) of breast cancer survivors [3, 4].

To manage the side effects of conventional cancer treatments and restore physical functioning and QOL, many female breast cancer survivors turn to complementary therapies such as Tai Chi and Qigong [5]. These mind-body exercise interventions simultaneously address physical and psychological needs and thus are particularly suitable for breast CA survivors [5]. 

Tai Chi (TC) Qigong, a mind-body exercise, was developed in China in the late 1970s and has gained popularity in both Eastern and Western countries [6, 7]. It is a simplified version of the traditional TC form and incorporates many Qigong training elements such as awareness of body movements and diaphragmatic breathing during practice. TC Qigong represents the essence of mind-body exercise [6, 7]. Training in TC Qigong has been reported to improve physical functioning and QOL in elderly people with chronic diseases [6, 8]. However, its potential for improving shoulder impairments and associated QOL in breast CA survivors is still not clear. To the best of our knowledge, only one study has investigated the effect of Tai Chi Chi Kung (TC Qigong) training on shoulder flexibility and handgrip strength in breast CA survivors [9]. Although the results were promising, the authors did not measure the functional mobility of the affected shoulder (e.g., hand-behind-the-back mobility) or shoulder muscular strength, which are crucial for many daily activities [10]. A study by Mustian and colleagues also reported that TC training could improve QOL in breast CA survivors, but they did not correlate QOL with any physical impairment [11]. 

The objectives of this study were (1) to compare the shoulder mobility and muscular strength among breast CA survivors with and without TC Qigong training with that of healthy individuals, (2) to compare the QOL of breast CA survivors with and without TC Qigong training, and (3) to explore the associations between shoulder impairments and QOL in breast CA survivors with regular TC Qigong training.

## 2. Methods

### 2.1. Sample Size Calculation

 Previous studies have shown that breast CA patients or survivors who exercise regularly have greater shoulder mobility and muscular strength when compared to control participants [12–14]. The reported effect sizes ranged from 1.2 to 3.1 for shoulder range of motion (ROM) [12] and from 0.7 to 1.3 for shoulder muscular strength [13]. According to a meta-analysis, the effect sizes for improving QOL outcome measures ranged from 0.2 to 1.0 [14]. In light of the scientific evidence, a relatively large effect size of 1.3 was expected for this study. Based on an alpha value of 5% and power of 80%, a sample of 22 breast CA survivors was needed.

### 2.2. Participants

Eleven women who had recovered from breast CA and received TC Qigong training at the Hong Kong Wushu and Art Service Centre, which provides TC Qigong training classes for cancer survivors, were recruited (TC Qigong CA group). In addition, age- and sex-matched control participants both with (CA-control group, *n* = 12) and without (healthy-control group, *n* = 16) history of breast CA were recruited from CA self-help groups and from the community, respectively. The inclusion criteria for the TC Qigong CA and CA-control participants were as follows (1) having a history of breast CA; (2) having received a mastectomy with or without adjuvant chemotherapy or radiotherapy; (3) having completed conventional breast CA treatments and being medically stable; (4) having no cognitive or psychological disorder (e.g., depression); (5) having no known neurological deficit resulting from breast CA treatments; (6) being aged 18 or older. An additional requirement for the participants in the TC Qigong CA group was that they had practiced the 18 Forms Tai Chi Internal Qigong for more than six months consecutively (three sessions per week, one hour per session). This practice comprises 18 movements designed to release tension in the body, promote relaxation, and increase awareness of breathing. Typical TC movements such as weight shifting, arm swinging, and punching and the gentle stretching of various body parts are performed smoothly with mental guidance and coordinated breath control [6]. The details of this TC Internal Qigong form have been described in Mak [7]. 

Individuals were excluded if they met any of the following criteria: (1) having significant neurological, musculoskeletal (e.g., history of shoulder dislocation or frozen shoulder), cardiovascular, peripheral vascular, or kidney disorders; (2) being receiving chemotherapy/radiotherapy, anti-cancer medication, acupuncture, or other cancer treatments; (3) having recurrent breast CA or cancer spread to other organs; (4) being exercising regularly; (5) having a smoking habit; (6) having received a lumpectomy instead of a mastectomy; or (7) being pregnant. The participants in the healthy-control group fulfilled the same aforementioned inclusion and exclusion criteria with the exceptions that they had no previous diagnosis of CA and hence had not received cancer treatment and had no previous experience in TC Qigong. 

Ethics approval was obtained from the Human Subjects Ethics Review Subcommittee of the administering institute. The study was explained to each participant and written informed consent was obtained from those who agreed to participate. 

### 2.3. Procedures

All of the assessment procedures were conducted in accordance with the Declaration of Helsinki and took place in the Sports Training and Rehabilitation Laboratory at the administering university. Standardized physical measurements were conducted by a registered physiotherapist who was blinded to the subject group. The demographic and QOL data were collected by a research assistant.

### 2.4. Outcome Measures

#### 2.4.1. Shoulder Mobility

A universal goniometer was used to measure the active ROM of shoulder flexion and abduction for the affected or the dominant arm (for bilateral mastectomy cases and healthy control participants). The ROM of the contralateral (unaffected) shoulder was not assessed because radiotherapy or chemotherapy can also affect the contralateral shoulder, making bilateral comparison unreliable [15]. Only active ROM was measured, as it is more functional than passive ROM [16]. 

The measurements were recorded while the participant was seated and the humerus was rotated externally through complete shoulder flexion and abduction. A standardized protocol was followed to minimize compensatory trunk movements or other trick movements [10]. All ROM measurements were for the affected shoulder complex (i.e., the scapula was not fixed) rather than for isolated movements of the glenohumeral joint. In addition, hand-behind-the-back mobility (i.e., the combined active ROM of scapular adduction and medial rotation, shoulder extension and internal rotation, elbow flexion, forearm pronation, wrist radial deviation, and finger extension) was assessed by measuring the distance (with a cloth measuring tape) between the spinous process of the seventh cervical vertebra and the tip of the middle finger as the participant reached up the back [10]. A shorter distance generally implies greater functional mobility in the affected upper limb. Hand-behind-the-back flexibility was examined because it is essential for performing many daily activities such as fastening a bra [10]. Shoulder ROM was measured three times in each movement direction and the highest value was used for analysis. Intrarater reliability for shoulder goniometric measurement was reported to be excellent in a previous study (ICC: 0.94, 95% CI: 0.91–0.99) [17].

#### 2.4.2. Shoulder Isokinetic Muscular Strength

All of the participants were first screened for contraindications of isokinetic testing according to the method described by Chan et al. [18]. The maximum strengths of the shoulder internal and external rotator muscles (primarily the rotator cuff muscles) of the affected upper limb or the dominant limb (for bilateral mastectomy cases and healthy control participants) were measured using a Cybex Norm isokinetic dynamometer (Computer Sports Medicine Inc., Stoughton, MA, USA). The shoulder rotator muscles were assessed because they dynamically stabilize the glenohumeral joint during many functional activities, and changes in strength may result in shoulder disorders [19]. In addition, the manipulation of the pectoral muscles (major internal rotator of the shoulder) and potential injury to the pectoral nerves during mastectomy may weaken the shoulder internal rotators, which may affect the QOL of breast CA survivors [20].

During the test, each participant laid on the testing couch in a crook-lying position. The participant's trunk was stabilized with straps and the nontested hand held onto the handle of the couch. The affected shoulder was positioned at 90° of abduction, elbow in 90° of flexion, and forearm in vertical, with the hand grasping the handle of the wrist/shoulder adapter (i.e., neutral starting position). The longitudinal axis of the humerus was aligned with the rotation axis of the dynamometer. The testing range for the external rotator muscles was from 70° internal rotation to 90° external rotation while the testing range for the internal rotator muscles was from 90° external rotation to 70° internal rotation [21]. An intermediate testing velocity of 180°/s was adopted because it is more functional and resembles the movement speed of many daily activities [22]. Familiarization trials were performed in the form of three submaximal and three maximal concentric shoulder internal/external rotator muscle contractions. After correcting for the effect of gravity on shoulder torque, five maximal concentric contractions of the shoulder internal and external rotator muscles were recorded as a test ensemble [21]. The average values of the five bodyweight-adjusted peak torques of both shoulder internal and external rotators were used for analysis.

#### 2.4.3. Quality of Life

QOL was assessed using the Functional Assessment of Cancer Therapy-Breast (FACT-B, version 4) scale. The reliability and validity of this instrument have been reported to be high [23, 24]. However, only the two CA groups completed the questionnaire because it is specifically designed for breast CA patients and survivors [23, 24]. 

The FACT-B includes 36 questions divided into five subscales: physical wellbeing (7 items), social/familial wellbeing (7 items), emotional well-being (6 items), functional well-being (7 items), and breast CA-specific concerns (9 items). These subscales comprise QOL-related statements that respondents rate on a 5-point Likert scale of agreement ranging from “not at all” (score 0) to “very much” (score 4). Item scores within a subscale were summed to produce a subscale score. The five subscale scores were then summed to obtain the total FACT-B score (i.e., item scores for all 36 items). A higher score indicates a more favorable QOL [23, 24].

### 2.5. Statistical Analysis

The Shapiro-Wilk statistic was first used to check the normality of the data. One way analysis of variance (ANOVA) was then used to compare the differences among the TC Qigong, CA-control, and healthy-control groups for the demographic and shoulder active ROM data and the isokinetic peak torques of the shoulder rotator muscles. Bonferroni tests were used to analyze the data post hoc as necessary. 

To compare the QOL variables between the TC Qigong CA and CA-control groups, a single multivariate analysis of variance (MANOVA) incorporating all of the FACT-B subscale scores was performed. The results from this analysis showed the effects of the group on all of the FACT-B subscale outcomes and the corresponding Bonferroni-adjusted *P* values, thus avoiding the increased probability of committing the type I errors associated with multiple comparisons. As the FACT-B total score was the sum of all subscale scores, an independent *t*-test was performed separately to compare this variable between the TC Qigong CA and the CA-control groups. Partial eta-squared and Cohen's *d*, which are the standardized measures of effect sizes for ANOVA and *t*-test, respectively, are also presented for the outcomes. By convention, partial eta-squared values of 0.01, 0.06, and 0.14 are considered to be small, medium, and large effect sizes, respectively, while Cohen's *d* values of 0.20, 0.50, and 0.80 are considered to be small, medium, and large, respectively [25]. 

If there were any significant between-group differences in the outcome measures, Pearson's product-moment correlation coefficient (Pearson's *r*) was used to explore the bivariate correlations between the physical impairment outcomes (i.e., shoulder ROM and muscular strength variables) and the QOL outcomes (i.e., FACT-B-derived variables) in the TC Qigong CA group. All of the statistical analyses were performed using SPSS version 20.0 and a significance level of 0.05 (two tailed) was chosen.

## 3. Results

 The characteristics of the participants are presented in Table 1. There were no significant differences between groups for any of the demographic variables (*P* > 0.05). Shoulder active ROM in flexion (*P* = 0.598), abduction (*P* = 0.964), and hand-behind-the-back (*P* = 0.464) directions were similar among the three groups (Table 2). However, significant between-group differences were found in the isokinetic peak torques of shoulder internal rotators (*P* < 0.025) and external rotators (*P* < 0.025). Post hoc tests revealed that participants in the TC Qigong CA group had significantly higher isokinetic peak torques (shoulder internal rotators, *P* = 0.049; shoulder external rotators, *P* = 0.040) than the CA-control participants. When compared to the healthy-control participants, the TC Qigong-trained breast CA survivors demonstrated no difference in shoulder isokinetic peak torques (*P* > 0.05), whereas the breast CA survivors without TC Qigong training showed significantly lower shoulder isokinetic peak torques (shoulder internal rotators, *P* = 0.033; shoulder external rotators, *P* = 0.025) (Table 2). None of the participants complained of shoulder pain or discomfort during the ROM and muscle strength assessments.

 Analysis of the FACT-B scores revealed no significant differences in the physical, social/familial, or emotional well-being subscale scores, or between the total FACT-B scores of the two CA groups (*P* > 0.05). However, the TC Qigong participants had higher functional well-being subscale scores (*P* = 0.012) and lower breast CA-specific concern subscale scores (*P* = 0.036) than the CA-control participants (Table 2). A further correlation analysis showed that only the FACT-B functional well-being subscale score was positively correlated with the isokinetic peak torque of shoulder internal rotators (*r* = 0.952, *P* < 0.001) and external rotators (*r* = 0.876, *P* < 0.001) in the TC Qigong CA participants.

## 4. Discussion

### 4.1. Shoulder Mobility

Our results demonstrate that all of our participants had a full range of shoulder motion in the flexion and abduction directions compared with the normative data [10] (Table 2). In addition, we found no significant difference in active shoulder ROM (flexion, abduction, and hand-behind-the-back directions) between breast CA survivors and healthy controls. This finding is in contrast to those of previous studies, which have reported restricted shoulder movements in breast CA survivors [12, 26]. A major reason for this discrepancy could be the differences in reporting shoulder ROM. Most previous studies compared the mobility of the affected shoulder to that of the unaffected shoulder and reported shoulder ROM deficits on the affected side [13, 26], whereas our study compared shoulder ROM in breast CA survivors to that of matched controls. Moreover, variations in postsurgical durations, the age ranges of participants, the treatments received, and the length of rehabilitation [15] might explain the inconsistent findings. The participants in our TC Qigong CA group had been recovering from breast CA for a prolonged period (average 6.8 years) and thus may have been fully rehabilitated and regained full shoulder mobility. TC Qigong training may not be able to increase shoulder flexibility. Certainly, a further prospective study would be necessary to confirm this postulation. 

### 4.2. Shoulder Isokinetic Muscular Strength

In agreement with Harrington et al., who reported reduced isometric shoulder strength, including internal and external rotator muscle strength, in breast CA survivors compared to healthy control participants [15], we also found that the body-weight-adjusted isokinetic peak torques of the shoulder internal and external rotators were lower in breast CA survivors (without TC Qigong training) than in the healthy control participants. Surgical trauma together with activity avoidance may be the major causes of shoulder muscle weakness [20, 27]. It seems that TC Qigong is a suitable exercise to improve shoulder muscular strength (Table 2) and therefore QOL in CA survivors [3, 4].

Although TC Qigong training focuses on relaxation and involves minimal muscle work, our results revealed that TC Qigong-trained participants had greater shoulder rotator muscle strength than the CA-control participants who had never received TC Qigong training. The body-weight-adjusted isokinetic peak torques of shoulder internal and external rotators in the TC Qigong-trained participants were actually comparable to those of the healthy control participants (Table 2). Coincidentally, Mustian et al. also reported improved hand grip strength in breast CA survivors after 12 weeks of TC Chi Kung (Qigong) training [9]. However, according to the exercise prescription guidelines of the American College of Sports Medicine, TC Qigong exercise involving free active upper limb movements does not meet the criteria for muscle strengthening (i.e., exercise close to volitional fatigue) [28]. Why might TC Qigong training improve upper limb muscle strength in breast cancer survivors? We postulate that the improvement is due to the mental training incorporated into this mind-body exercise. TC Qigong practitioners use the mind to control body movements during practice. There is evidence that mental control of muscle contractions can enhance the cortical output signals that drive the “mentally trained muscles” to a higher activation level and thus increase muscular strength [29]. Moreover, although the exercise intensity of TC Qigong appears low, ROM exercises may prevent muscle atrophy and reduce scar fibrosis and hence attenuate significant loss of muscular strength [30].

### 4.3. Quality of Life

Another encouraging finding was that the scores on the FACT-B functional well-being subscale in the TC Qigong CA group were significantly higher than those of the CA-control group (Table 2). This result is consistent with that of Oh et al. [31], who used a randomized controlled study design and found that the FACT functional well-being subscale scores of cancer patients improved significantly and were higher than those of the control participants after 10 weeks of Qigong training. However, their study did not offer any explanation of the patients' improved daily functioning such as the ability to work and enjoy life after Qigong exercise [31]. We attempted to provide such an explanation through correlation analysis and our results suggest that higher shoulder rotator muscle strength was associated with higher FACT-B functional well-being subscale scores in the TC Qigong participants. This finding is logical because the shoulder rotators, including the rotator cuff muscles, are the major dynamic stabilizers of the glenohumeral joint during many daily functional activities. Decreases in the strength profile have been found to result in shoulder disorders, functional disability, and reduced QOL in patient populations [19, 32]. 

 Despite the potential positive effect of TC Qigong training on functional well-being in breast CA survivors, the exercise may not relieve survivors' concerns about breast CA-related problems. Our results reveal that the breast CA survivors who participated in TC Qigong training experienced more side effects of conventional cancer treatments (e.g., swollen arm), and their psychological status (e.g., self-esteem relating to sexual attractiveness), as reflected by the FACT-B breast CA-specific concerns score (Table 2), was inferior to that of the CA-control group participants. These findings are quite different from those of a previous study that reported improved self-esteem and health-related QOL in breast CA survivors after TC Chi Kung (Qigong) training [11]. Because this is a cross-sectional study, it is uncertain whether the negative findings were due to the ineffectiveness of TC Qigong exercise or due to sampling (self-selection) bias. Perhaps those breast CA survivors who had greater concerns about their physical and psychological health were keener to participate in TC Qigong training. Thus, this group of participants might be more sensitive to the negative changes related to cancer treatments. Randomized controlled studies may be necessary to confirm the effects of TC Qigong training on FACT-B breast CA-specific concerns.

Regarding the physical, social/family, and emotional well-being subscale scores, no between-group differences were observed in this study (Table 2). Again, these findings are quite different from those of Oh et al., who discovered the positive effects of Qigong training on the QOL of CA patients [31]. We conjecture that our insignificant findings were due to our relatively small sample size. Indeed, the effect sizes of these three subscale scores ranged from moderate to large (0.033–0.153, resp.). Further investigation using a larger sample might detect between-group differences.

As no between-group differences were found for most of the FACT-B subscale scores, the total FACT-B score, which is the sum of all subscale scores, was similar between the two groups (Table 2). This implies that, overall, participants in the TC Qigong CA group had a QOL that was similar to that of those in the CA-control group. Our findings disagree with those of a previous study that reported improved QOL in breast CA patients after TC Chi Kung (Qigong) training [11]. Further prospective studies using larger samples are necessary to confirm the results. 

### 4.4. Study Limitations and Recommendation for Future Research

 TC Qigong training appears to improve shoulder rotator muscle strength and functional well-being in breast CA survivors. However, due to the cross-sectional research design and relatively small sample size, we cannot confirm the results without larger-scale, randomized controlled clinical trials. Moreover, some of our participants could not recall the exact type of surgery (e.g., radical or modified mastectomy) they had received and we were unable to retrieve their medical records. Exercise history was also not documented in this study. All these factors may confound the results. Further study may include a more homogenous sample and request the participants to present their surgical or medical records and exercise history during the screening process.

## 5. Conclusions

Although there was no obvious impairment of shoulder mobility, impairment of shoulder rotator muscle strength was apparent among the breast CA survivors. Tai Chi Qigong training might improve shoulder muscular strength and, therefore, the functional well-being of breast CA survivors. TC Qigong can be considered as a potential therapeutic intervention for long-term breast CA survivors.

## Figures and Tables

**Table 1:** Characteristics of participants (mean ± SD).

	TC Qigong CA group (*n* = 11)	CA-control group (*n* = 12)	Healthy-control group (*n* = 16)	*P*
Age (year)	58.3 ± 10.1	53.8 ± 4.2	56.8 ± 6.4	0.304
Height (cm)	155.5 ± 4.3	156.7 ± 6.0	156.5 ± 5.6	0.859
Weight (kg)	50.4 ± 7.4	55.6 ± 8.8	57.2 ± 5.5	0.063
Body mass index (kg/m^2^)	20.8 ± 3.0	22.6 ± 3.3	23.3 ± 1.2	0.054
Breast CA affected/surgical side (*n*)	Left = 6; Right = 4; Bilateral = 1	Left = 10; Right = 2	N/A	—
Mastectomy (*n*)	11	12	0	—
Postmastectomy duration (year)	6.8 ± 4.3	7.2 ± 4.0	N/A	0.843
Radiotherapy (*n*)	11	12	0	—
Chemotherapy (*n*)	2	1	0	—
Qigong experience (year)	0.9 ± 0.2	0	0	—

**Table 2:** Comparison of outcome measures between groups (mean ± SD).

	TC Qigong CA group (*n* = 11)	CA-control group (*n* = 12)	Healthy-control group (*n* = 16)	Effect size	*P*
Shoulder active ROM (affected side/dominant side)					

Flexion (degree)	178.2 ± 4.6	176.8 ± 6.8	178.8 ± 3.4	0.028	0.598
Abduction (degree)	179.1 ± 3.0	179.1 ± 2.9	179.3 ± 2.5	0.002	0.964
Hand behind back (cm)	8.7 ± 6.3	6.0 ± 4.5	8.1 ± 5.8	0.042	0.464

Body-weight-adjusted isokinetic peak torque at 180°/s (affected side/dominant side)					

Shoulder internal rotators (Nm)	26.7 ± 5.4^†^	19.9 ± 7.0	26.5 ± 6.6^†^	0.198	0.019**
Shoulder external rotators (Nm)	27.8 ± 5.3^†^	21.7 ± 6.4	27.7 ± 5.2^†^	0.210	0.014**

FACT-B					

Physical wellbeing	24.6 ± 5.2	22.5 ± 6.3	N/A	0.033	0.406
Social/family wellbeing	18.5 ± 2.4	14.8 ± 5.9	N/A	0.153	0.065
Emotional wellbeing	20.6 ± 2.5	18.2 ± 4.9	N/A	0.089	0.167
Functional wellbeing	25.6 ± 2.5	21.2 ± 4.7	N/A	0.264	0.012*
Breast CA-specific concerns	25.7 ± 5.0	30.3 ± 4.9	N/A	0.193	0.036*
Total score	114.9 ± 10.3	107.0 ± 16.0	N/A	0.587	0.179

* Denotes a significant difference at *P* < 0.05.

** Denotes a significant difference at *P* < 0.025.

^†^ Denotes a significant difference at *P* < 0.05 when compared with the CA-control group.
